# Supplementary material for: Understanding Patient Evaluation of Abnormal Uterine Bleeding (AUB): A Standardized Patient Case on AUB for OB/GYN Clerkship Students
Source: MedEdPORTAL. 2022 Jan 28;18:11216. doi: 10.15766/mep_2374-8265.11216 (PMC8795174; doi:10.15766/mep_2374-8265.11216)
Supplement: Supplementary file 1 — SP Information.docxLearner Information.docxPostencounter Learner Note.docxPostencounter SP Evaluation.docxLearner End-of-Clerkship Feedback.docx [file mep_2374-8265.11216-s001.zip › A. SP Information.docx]

Appendix A: Pre-Encounter SP Information

MedEdPORTAL Standardized Patient Case Development Tool

Date: June 2018

Primary Case Author: Sarah Dotters-Katz, MD, MMHPE

Standardized Patient Educator: Tracey Reynolds, BA

Name of Case: “I bleed all the time”

Name of educational and or assessment activity: Clinical Practice Examination

Patient Name: Joanne Davis (she/her/hers)

Chief Complaint: Vaginal bleeding

Most likely Diagnosis and Differential with rationale from history and/or physical exam:

| Most likely diagnosis: Endometrial Hyperplasia / Malignancy | Heavy, intermenstrual vaginal bleeding |
| --- | --- |
|  | BMI 40 kg/m^2^ |
|  | Early menarche (age 10) |
|  | Age >35 |
|  | Slightly enlarged uterus on exam (exam limited by body habitus) |
| Diagnosis #2: Adenomyosis | Heavy, prolonged vaginal bleeding |
|  | Mild to moderate abdominal cramping |
|  | Slightly enlarged uterus on exam (exam limited by body habitus) |
| Diagnosis #3: Endometrial Polyp | Heavy, intermenstrual vaginal bleeding |
|  | BMI 40 kg/m^2^ |
| Diagnosis #4: Uterine Leiomyoma | Heavy vaginal bleeding |
|  | BMI 40 kg/m^2^ |
|  | History of cesarean section for breech presentation (possible submucosal leiomyoma affecting fetal presentation) |

Challenge question/moment:

If/when student asks about sexual history, SP will state “My husband doesn’t want to have sex with me because I am bleeding all the time. He’s grossed out by it; I don’t blame him, but it makes me feel bad.”

Domains: Check all that apply

- Professionalism

X Communication and Interpersonal skills

X Medical History

X Physical exam

- Shared Decision Making

X Patient Education

X Clinical Reasoning

X Documentation

- Handoff
- Presentation
- Other:

Type and level of learner: Health professional student (i.e., medical, nursing) in their clinical year

Case Objectives: please list specific objectives for each of the domains you have checked above:

1. The student will obtain a complete problem focused history as it pertains to gynecologic symptoms
2. The student will obtain a complete gynecologic history as well as sexual history
3. The student will develop a complete differential diagnosis for abnormal uterine bleeding
4. The student will develop and begin the work-up to narrow the diagnosis
5. The student will counsel the patient regarding next steps and management of abnormal uterine bleeding

| SETTING: outpatient, in patient, ED, home, nursing home, rehab, group etc. | Outpatient, new patient encounter |
| --- | --- |
| PATIENT PROFILE: Information about the “patient” that helps select an SP and helps the learner get an understanding of them as a person. SP will know more information about the patient than learner will ever ask but allows SP to portray a fully developed patient personality. If none of the items below are particulars for the case please write “all may be used.” | |
| Age range | >35 – early 40’s |
| Religious/spiritual background | All may be used |
| Sex (e.g., male, female, intersex, transwoman, transman) | Female |
| Sexual Orientation (e.g., heterosexual, lesbian, gay, bisexual, pansexual, queer, asexual) | Heterosexual |
| Gender expression (e.g., man, woman, gender queer) | Woman (she/her/hers pronouns) |
| Race/ethnicity: | All may be used |
| Physical description (e.g., BMI, height range) | BMI 40 kg/m^2^ |
| Physical limitations | None are mentioned in the case, but an SP with any physical limitations may portray this character |
| Patient appearance (e.g., disheveled, hospital gown, business casual, casual) | Appears worried, wearing an examination gown with appropriate undergarments (i.e., shorts, tank top) under gown. |
| Moulage + location (e.g., none, bruises, scars, body piercing, tattoos) | None |
| Affect (e.g., pleasant, cooperative) | Mood is not great, but she is not depressed. She is worried about symptoms and hoping to be taken seriously. |
| Family group (e.g., who is family, who they live with) | Lives with husband and 3 children (12 years old, 7 years old, 4 years old) |
| Education | High school diploma |
| Level of health literacy | Middle—is familiar with anatomical words such as uterus or ovary |
| Employment, if any - present and past, noting any current stresses | Works at Wal-Mart |
| Home/homeless - type of dwelling, number of stories, owned or rented | Rents 2-bedroom, 2-bathroom home |
| Financial situation- any current stresses | Low class, but no current stresses |
| Insurance Status (e.g., un/under/insured, public/private, HMO/PPO) | Private insurance through her husband’s work |
| Habits (i.e., diet, exercise, caffeine, smoking, alcohol, drugs) | Tobacco: Denies  Alcohol, drugs, substances: Does not use drugs. Uses alcohol only on special occasions, 1-2x/month  **If asked – all alcohol abuse related questions negative  Diet: Eats fast food a lot, tries to add in vegetables, but doesn’t really like them.  Exercise: None |
| Activities (i.e., hobbies, sports, clubs, friends) | All may be used |
| Typical day - what is the usual daily routine | Wakes up between 6-7am to get the kids ready for school, breakfast generally on the go, arrives to work at 8 am. Returns home after 5:30pm after picking the kids up from their after school activities. Helps children with homework until her husband gets home at 6:30pm. They eat dinner together (most nights fast food) and usually watch a TV show before going to bed. |

| CASE INFORMATION | |
| --- | --- |
| Chief Concern: What the patient will say when greeted by the student. The patient’s primary reason for seeking medical care often stated in his/own words. | “I bleed all the time” |
| Additional Concerns: Other, if any, concerns the patient has today (i.e., symptoms, requests, expectations, etc.) that will become part of set agenda. | None |
|  | |
| THE PATIENT STORY: The SP will be asked to tell their symptom story and the personal and emotion impact for each of their concerns. You will want to write this is the patient voice. The symptom story should be able to answer this question: “Tell me more about [chief concern/additional concern], starting at the beginning and bringing me up to now.”  The personal context should be able to answer questions concerning the broader personal/psychosocial context of symptoms, especially the patient beliefs/attributions.  The emotional context should be able to ask how are you doing with this, how does this make you feel, how has this affected you emotionally? IMPACT: How has this affected your life? How has this been for your family? | “Well, my periods have always been on the heavier side, especially since I had my last baby 4 years old. But, they have gotten worse over the last 6 months I would say. Some days I bleed through 6 super tampons and a super pad (combination) in a day! I’m always afraid to stand up after sitting for a prolonged period of time. It’s absolutely horrible. It has negatively impacted my confidence, sex life, and day-to-day activities—especially at work. I’m constantly worrying ‘am I going to bleed through another pair of underwear or pants today?’ I’ll be happy when this is all behind me.” |
| HISTORY OF PRESENT ILLNESS: Although some of the HPI will be given in the patient’s symptom story, the learners will expand the story during the direct question section. Below describe the detailed history, usually about the chief concern, which the student must develop in order to make a useful assessment of the problem: | |
|  | |
| Onset (when; gradual or sudden) | About 4 years ago I started having heavier periods after my last delivery, but I noticed a progression in the amount of blood I would experience about 6 months ago |
| Setting (what was going on or where was patient when symptoms first noticed?) | About 4 years ago after my last delivery |
| Duration (how long) | It feels like I bleed every day. There are about 5 heavy days, but that seems to happen more than once a month. Other days in the month I usually need 1 regular pad for light bleeding or spotting. |
| Time relationships (frequency, constant or intermittent) | Feels like I am bleeding or spotting every day; like my period never stops. |
| Location | I am sure the blood is coming from my vagina |
| Radiation | None |
| Quality | It’s horrible for my quality of life. I have to wear a super tampon plus a super pad on the heavy days, that’s at least 5 days in the month. I bleed through underwear and pants. Sometimes there are clots. |
| Amount | It is really getting bothersome. |
| Aggravated by what | Nothing that I can tell. |
| Relieved by what | Motrin seems to help on the bad days when I have cramps, which usually align with the heavier 5 days of bleeding. |
| Associated with what | I have mild to moderate cramping a few days a month. I have not noticed any pain when I urinate. I don’t think my poop has changed with the increase in bleeding. I also have not noticed any pain or increased bleeding with sex. |
| Attitude (what does the patient think is the problem, and how does he/she feel about it) | The ongoing bleeding is really bothersome. It seems like so much sometimes; I worry it may be something serious. |
| Overall course | Noticed more heavier periods after the birth of my last child, but it has gotten progressively worse over the past 6 months; when I see clots I get worried. |
| REVIEW OF SYSTEMS: Significant positives and negatives | |
| *Negative* | *Positive* |
| No fevers or chills | Vaginal bleeding, sometimes with clots |
| No GI symptoms over the course of the month/no change in BM with menstrual cycle | Mild to moderate lower abdominal pain and cramping |
| No UTI symptoms (no pain or burning with urination, no urinary frequency or urgency |  |
| No heat or cold intolerance |  |
| No skin, hair or nail changes |  |
| No unintentional weight changes |  |
|  | |
| Past medical history |  |
| Medication allergies (Name and reaction) | Motrin 600mg as needed |
| Environmental allergies (Name and reaction) | None |
| Illnesses | None |
| Vaccinations | Up to date, had flu shot this year |
| Surgeries | Delivery of 1 child by cesarean for breech presentation, elective 1^st^ trimester D&C for therapeutic abortion |
| Accidents/ injuries/ trauma | None |
| Hospitalization | Only for birth of children |
|  | |
| Inclusive sexual and reproductive history | |
| Sexual practices  Sexual partners  Protection: Use of safer sex practices  Use of birth control if appropriate  Risk of intimate partner violence | Vaginal and oral sex; does orgasm, currently dissatisfied with sex life due to ongoing vaginal bleeding  Currently 1 male partner, lifetime 3 male partners  None, “It’s just me and my husband”  Never used hormonal birth control, only condoms.  None, no domestic violence no sexual abuse |
| Ob/GYN HISTORY | Age of onset of menses: 10  Periods: Used to be regular, every 28 days, lasting 5-6 days, heavy first 3 days. Now pretty persistent. Seems to have had a continuous period for at least 6 months, with heavier bleeding with clots at least 5 days out of the month. The rest of the month is a mix of light flow and spotting mostly every day.  Age of menopause: N/A, mother went through menopause at ~age 53  Pap smears: 1 abnormal Pap in 2005, but they have been ok since. Pap smear last year was normal and the additional testing (HPV) was negative.  Hx of STDs: Chlamydia as a teenager.  Number of pregnancies: 4  Number of live births: 3  Number of miscarriages: 0  Number of abortions: 1 |
| Medications | Prescription/dose/reason: None  Over the counter/dose/reason: Motrin PRN/600mg/cramps  Herbs/supplements/dose/reason: None  Other: None |
| Immunizations | X Tetanus  X Flu  X Hepatitis   - Pneumovax - HPV - Other |
| Tobacco products:   - Cigarettes - Cigar - Pipe - Chew - E-cigarettes | X Never   - Past- year started/year quit - Current   - Quantity   - # of years |
| Alcohol   - Beer - Wine - Liquor - Other | - Never - Past- year started/year quit   X Current   - - Quantity: 1-2 glasses of wine month/special occasions   - # of years: Past 15 years |
| Drugs   - Weed - Cocaine - Heroin - Meth - Other - IV - Inhalants - Other | X Never   - Past- year started/year quit - Current   - Quantity - # of years |
| Diet (describe) | Primarily fast food, tries to add in vegetables, but doesn’t really like them. |
| Exercise (describe) | None |
| List any other important social history or information important to this case | None |
| Family history |  |
| Mother, Father, Siblings, Grandparents, and other significant findings. | Family member, status (living age/deceased), medical conditions   - Father, living 60 years old, type 2 diabetes, hypertension - Mother, living 62 years old, type 2 diabetes - Sibling, living 40 years old, Hypertension - Children, 12-, 7-, 4-years old, healthy |
|  |  |
| Physical Exam- List exam maneuvers expected for this case and any abnormal findings that SP will simulate. (tenderness, hyper-hypo reflex, rebound, weakness etc.)  N/A | |
| PHYSICAL EXAM FINDINGS |  |
| 1. Written in layman’s terms | N/A |
| 1. General appearance- affect, appearance, position of patient at opening (i.e. sitting, laying down, holding abdomen etc.) | Well appearing female sitting up and in no acute distress but appears anxious and worried. |
| 1. Vital signs | Normal vitals, BP on higher end 135/85 (normal for her) |
| 1. Specific findings and affect | Student needs to ask to perform pelvic exam – **Finding’s card** with results: normal female external genitalia, blood in vault, some blood at external os, uterus anteverted and slightly enlarged but exam limited by body habitus, no adnexal masses |
| 1. Response to certain physical movements | N/A |
|  |  |
| DIAGNOSIS AND DIFFERENTIAL |  |
| Diagnosis with support from positive and negative history and PE findings | Endometrial hyperplasia/malignancy: Heavy, prolonged and intermenstrual vaginal bleeding, BMI 40 kg/m^2^, early menarche (age 10), age >35 years old, no history of hormonal contraceptive use |
| Differential with support from positive and negative history and PE findings | Adenomyosis: Heavy, prolonged vaginal bleeding, mild to moderate abdominal cramping improved with Motrin, slightly enlarged uterus on exam (exam limited by body habitus)  Endometrial Polyp: Heavy, prolonged vaginal bleeding, BMI 40 kg/m^2^, borderline high/normal blood pressure,  Uterine Leiomyoma: Heavy vaginal bleeding, BMI 40 kg/m^2^, slightly enlarged uterus on exam, history of cesarean section for breech presentation (possible mucosal or submucosal affecting fetal rotation).  Ovulatory Dysfunction: Heavy, intermenstrual vaginal bleeding  Coagulation Disorder: Heavy, intermenstrual vaginal bleeding, however unlikely due to no other signs or history of deep or superficial bleeding |
| MANAGEMENT OR DIAGNOSTIC PLAN | Labs: CBC, TSH, FSH, pregnancy test, Coagulation studies (PT/PTT/INR)  Imaging: Pelvic Ultrasound  Procedures: consider endometrial biopsy  Counseling: discuss top diagnoses while highlighting the need for further evaluation via labs and imaging. Explain step by step management plan and follow-up.  Treatment: discuss the need to postpone definitive treatment until further investigation occurs. |
| PROFESSIONALISM ISSUES OR CHALLENGES: | Student should demonstrate empathy to patient complaint and provide supportive statements when SP discloses impact on quality of life, anxiety regarding potential diagnosis, and sex life with husband. |
