# Supplementary material for: Understanding Patient Evaluation of Abnormal Uterine Bleeding (AUB): A Standardized Patient Case on AUB for OB/GYN Clerkship Students
Source: MedEdPORTAL. 2022 Jan 28;18:11216. doi: 10.15766/mep_2374-8265.11216 (PMC8795174; doi:10.15766/mep_2374-8265.11216)
Supplement: Supplementary file 1 — SP Information.docxLearner Information.docxPostencounter Learner Note.docxPostencounter SP Evaluation.docxLearner End-of-Clerkship Feedback.docx [file mep_2374-8265.11216-s001.zip › C. Postencounter Learner Note.docx]

Standardized Patient CPX Student Scoring Criteria: Abnormal Uterine Bleeding

*Format adapted from Hagey et al^1^*

**Post-Encounter Learner Note**

HISTORY: Describe the history you just obtained from this patient. Include only information (pertinent positives and negatives) relevant to this patient's problem(s).

**Scoring (0.5 points for each)**

1. Chief Complaint
   1. Location: vaginal bleeding
   2. Severity: heavy, horrible for quality of life
   3. Onset/Duration: heavy since last baby, but worse 6 months ago, feels like every day, sometimes really heavy (5d/month)
   4. Last menstrual period: hard to tell
   5. Associated symptoms: no vaginal discharge, no urinary symptoms, no diarrhea/constipation
   6. Alleviating factors: Motrin helps on bad days
2. Medications: none besides occasional Motrin
3. OB History: 3 kids, 2 vaginal, 1 cesarean, 1 abortion
4. GYN/Sexual History: distant history of abnormal pap smear, chlamydia as a teen
5. Past medical history and Social history: none relevant to case
6. ROS: negative

PHYSICAL EXAMINATION: Describe any positive and negative findings relevant to this patient's problem(s). Be careful to include only those parts of examination you performed in this encounter.

**Scoring (0.5 points for each)**

1. Vitals/general appearance
2. Abdominal Exam
3. Pelvic exam – student must request

DATA INTERPRETATION: Based on what you have learned from the history and the physical examination, list up to 3 diagnoses that might explain this patient's complaint(s). List your diagnoses from most to least likely. For some cases, fewer than 3 diagnoses will be appropriate. Then, enter the positive or negative findings from the history and the physical examination (if present) that support each diagnosis.

**Scoring (1 point for diagnosis, 1 point for supporting evidence)**

1. Uterine Polyp
2. Uterine leiomyomata
3. Endometrial cancer/hyperplasia
4. Ovulatory dysfunction
5. Adenomyosis

DIAGNOSTIC STUDIES/MANAGEMENT PLAN: Based on your differential diagnosis, list initial diagnostic studies (if any) you would order for each listed diagnosis (e.g. restricted physical exam maneuvers, laboratory tests, imaging, ECG, etc.). You may also list any management plan you would offer to the patient based on your differential.

**Scoring (1 point for each)**

1. Pelvic exam
2. bHCG
3. Pelvic ultrasound
4. Endometrial biopsy
5. CBC
6. TSH
7. Coagulation panel
