# Supplementary material for: Understanding Patient Evaluation of Abnormal Uterine Bleeding (AUB): A Standardized Patient Case on AUB for OB/GYN Clerkship Students
Source: MedEdPORTAL. 2022 Jan 28;18:11216. doi: 10.15766/mep_2374-8265.11216 (PMC8795174; doi:10.15766/mep_2374-8265.11216)
Supplement: Supplementary file 1 — SP Information.docxLearner Information.docxPostencounter Learner Note.docxPostencounter SP Evaluation.docxLearner End-of-Clerkship Feedback.docx [file mep_2374-8265.11216-s001.zip › D. Postencounter SP Evaluation.docx]

Standardized Patient CPX Student Scoring Criteria: Abnormal Uterine Bleeding

*Format adapted from Hagey et al*

**Post-Encounter Standardized Patient Evaluation**

**Interpersonal**

| Interpersonal skills reflect a student’s ability to provide an effective exchange of information and develop a therapeutic relationship with their patients. They are a combination of communication skills (questioning and information-sharing) and relational skills (empathy and respect). The components of interpersonal skills being evaluated in this exam are: | | | | | |
| --- | --- | --- | --- | --- | --- |
| 1.  Introduction | (1)  Poor: *Does not introduce self *Does not identify you by name *Disinterested greeting | (2)  Fair | (3)  Adequate: *Introduces self *Identifies you by name *Appropriate greeting | (4)  Very Good | (5)  Excellent: *Introduces self by name and position *Identifies you by name *Warm and engaging greeting |
| 2.  Questioning Skills | (1)  Poor: *No use of open-ended questions *Multiple interruptions *Scattered and disjointed line of questioning | (2)  Fair | (3)  Adequate: *Some use of open-ended questions *Few interruptions *Basic flow to line of questioning | (4)  Very Good | (5)  Excellent: *Effective use of multiple open-ended questions *Zero to minimum interruptions *Smooth transitions and natural flow |
| 3.  Elicit Patient Perspective | (1)  Poor: *Uninterested in your explanatory model *Unconcerned with how illness may affect well-being *Resistant to incorporating your viewpoints into decision making | (2)  Fair | (3)  Adequate: *Acknowledges your explanatory model *Receptive to your concerns over impact of illness on well-being *Incorporates your input into decision making | (4)  Very Good | (5)  Excellent: *Proactively assesses your explanatory model *Explores your concerns over impact of illness on well-being *Proactively solicits your viewpoint in decision making |
| 4.  Verbal Communication | (1)  Poor: *Uses medical jargon excessively *Thoughts consistently disorganized *Tone of speech conveys indifference or detachment | (2)  Fair | (3)  Adequate: *Limited use of medical jargon *Most thoughts are well organized *Comfortable tone of speech | (4)  Very Good | (5)  Excellent: *Avoids medical jargon or readily explains it *Thoughts are consistently well organized and easy to understand *Uses warm and accepting tone of speech |
| 5.  Non-verbal Communication | (1)  Poor: *Unable to make eye contact *Awkward physical distance, facial expressions, or touching *Conveys disinterest or apathy | (2)  Fair | (3)  Adequate: *Maintains some eye contact *Appropriate physical distance, facial expressions, or touching *Conveys interest and concern | (4)  Very Good | (5)  Excellent: *Consistent eye contact *Uses physical distance, facial expressions, or touching effectively *Conveys attentiveness and compassion |
| 6.  Empathy | (1)  Poor: *Ignores or fails to detect emotional cues *Empathetic responses/emotional support absent or forced *Dismissive of pain or anxiety | (2)  Fair | (3)  Adequate: *Responds to emotional cues *Provides empathetic responses/emotional support *Acknowledges pain or anxiety | (4)  Very Good | (5)  Excellent: *Perceptive of emotional cues and encourages emotional expression *Provides empathetic responses/emotional support with genuineness and sincerity *Attentive to pain or anxiety |
| 7.  Respect | (1)  Poor: *Judgmental attitude *Makes you feel inferior *Physical exam without regard to pain or modesty | (2)  Fair | (3)  Adequate: *Non-judgmental attitude *Treats you as equal *Physical exam respectful of pain and modesty | (4)  Very Good | (5)  Excellent: *Accepting attitude *Establishes partnership *Physical exam with great sensitivity to pain and modesty |
| 8.  Closure | (1)  Poor: *No explanation of impression or plan *No inquiry into remaining questions *No cordial closing remarks | (2)  Fair | (3)  Adequate: *Explains impression and plan *Inquires about remaining questions *Cordial closing remarks | (4)  Very Good | (5)  Excellent: *Thorough discussion of impression and plan *Seeks unanswered questions, verification of understanding, and comfort level *Warm and grateful closing remarks |

**Comments**

| 9.  Student Feedback: |
| --- |
