# Supplementary material for: Understanding Patient Evaluation of Abnormal Uterine Bleeding (AUB): A Standardized Patient Case on AUB for OB/GYN Clerkship Students
Source: MedEdPORTAL. 2022 Jan 28;18:11216. doi: 10.15766/mep_2374-8265.11216 (PMC8795174; doi:10.15766/mep_2374-8265.11216)
Supplement: Supplementary file 1 — SP Information.docxLearner Information.docxPostencounter Learner Note.docxPostencounter SP Evaluation.docxLearner End-of-Clerkship Feedback.docx [file mep_2374-8265.11216-s001.zip › E. Learner End-of-Clerkship Feedback.docx]

**Appendix E – Learner End-of-Clerkship Feedback**

1. Please rate the educational value of the following Ob/Gyn clerkship experiences:

*(1=poor, 2=below average, 3=average, 4=above average, 5=excellent)*

- 1. Pelvic teaching session
  2. Pelvic Anatomy Teaching Session
  3. Work/Attending Rounds
  4. Formal Student Presentation
  5. Morbidity and Mortality conference
  6. Ob/Gyn Grand Rounds
  7. Faculty Case Based Learning
  8. Standardized Patient Encounters

1. Please rate how well the Standardized Patient Encounters enhanced your learning on this clerkship:

*(1=not at all, 2=* *Less than adequate, 3=* *Adequately well, 4*= More than adequate*, 5=* Extremely well*)*

1. Did you receive feedback from clerkship faculty member about your Standardized Patient Encounter performances?

*(yes or no)*

Please provide any additional comments:

|  |
| --- |
