# Supplementary material for: Understanding Patient Evaluation of Abnormal Uterine Bleeding (AUB): A Standardized Patient Case on AUB for OB/GYN Clerkship Students
Source: MedEdPORTAL. 2022 Jan 28;18:11216. doi: 10.15766/mep_2374-8265.11216 (PMC8795174; doi:10.15766/mep_2374-8265.11216)
Supplement: Supplementary file 1 — SP Information.docxLearner Information.docxPostencounter Learner Note.docxPostencounter SP Evaluation.docxLearner End-of-Clerkship Feedback.docx [file mep_2374-8265.11216-s001.zip › B. Learner Information.docx]

**Appendix B: Pre-Encounter Learner Information**

*Format adapted from Hagey et al*

Instructions to the Student:

**Patient Information**

| **Patient Name:**Joanne Davis  **Setting:**  Outpatient clinic  **Patient Information:**  Ms. Davis is a 37-year-old female who has presented as a new patient to your clinic for vaginal bleeding  **Vitals:**  Blood Pressure:            135/85  Pulse:                            80  Temperature:               98.6C  Respiration:                  20 |
| --- |

**Your Task**

| In the ***15 minutes*** with the patient:  Obtain problem-focused history that includes and Ob/Gyn history.  Perform a focused physical exam.  In the remaining ***10 minutes***:  Write a note that includes your medical decision-making, including a prioritized differential and a proposed workup/management plan? |
| --- |
