# Supplementary material for: Understanding Patient Evaluation of Abnormal Uterine Bleeding (AUB): A Standardized Patient Case on AUB for OB/GYN Clerkship Students
Source: MedEdPORTAL. 2022 Jan 28;18:11216. doi: 10.15766/mep_2374-8265.11216 (PMC8795174; doi:10.15766/mep_2374-8265.11216)
Supplement: Supplementary file 1 — SP Information.docxLearner Information.docxPostencounter Learner Note.docxPostencounter SP Evaluation.docxLearner End-of-Clerkship Feedback.docx [file mep_2374-8265.11216-s001.zip › Educational_Summary_Report_11216.pdf]

MedEdPORTAL® The AAMC Journal of Teaching and Learning Resources

Original Publication Open Access

Understanding Patient Evaluation of Abnormal Uterine Bleeding (AUB): A Standardized Patient Case on AUB for OB/GYN Clerkship Students

LaMani D. Adkins*, Benjamin S. Harris, MD, MPH, Cescille Gesher, Tracey Reynolds, Kelly Branford, Melody Baldwin, MD, MPH, Sarah Dotters-Katz, MD, MMHPE

*Corresponding author: ladkinsmed@gmail.com

Abstract

Introduction: The differential diagnosis for abnormal uterine bleeding (AUB) among reproductive-age women is broad and includes common and life-threatening conditions. Recognition and accurate diagnosis of AUB are important but can be challenging for medical students. We developed a standardized patient (SP) encounter for medical students during their OB/GYN clerkship. **Methods:** We implemented two SP encounters, on AUB and dyspareunia, that included a postencounter note and SP evaluations. Here, we describe the implementation of the SP encounter on AUB. Students received formative feedback on their interpersonal and history-taking skills, differential diagnosis, and management plan from the SP as well as OB/GYN residents and faculty. Student cumulative feedback was obtained mid-clerkship and following the clerkship. Summary statistics and qualitative data for students' experiences are reported. **Results:** SP cases were implemented at the Duke University School of Medicine with 101 second-year medical students who completed the encounter from September 2018 to July 2019. Regarding the AUB case, SPs identified students as adequate history takers, with a mean evaluation score of 3.45 ($SD = 0.15$) out of 5. Most students (94%) correctly identified at least one diagnosis and provided evidence. Endometrial cancer/hyperplasia (63%) and uterine leiomyoma (60%) were most likely to be identified. Regarding both SP encounters, of the 82 students (81%) completing the end-of-clerkship survey, 57% indicated that the experience enhanced their overall learning at least adequately well or better. **Discussion:** The AUB case provided students with the opportunity to exercise their diagnostic and management skills.

Keywords
OB/GYN Clerkship, Abnormal Uterine Bleeding, Clinical Skills Assessment/OSCEs, OB/GYN, Standardized Patient

Educational Objectives

By the end of this activity, learners should be able to:

1. Obtain a problem-focused history as it pertains to abnormal uterine bleeding.
2. Obtain a complete gynecologic history, including a sexual history.
3. Develop a differential diagnosis including at least one correct diagnosis of abnormal uterine bleeding with supporting historical evidence.
4. Recommend an initial workup of abnormal uterine bleeding based on proposed differential diagnoses.

Citation:
Adkins LD, Harris BS, Gesher C, et al. Understanding patient evaluation of abnormal uterine bleeding (AUB): a standardized patient case on AUB for OB/GYN clerkship students. *MedEdPORTAL*. 2022;18:11216.
https://doi.org/10.15766/mep_2374-8265.11216

Introduction

Abnormal uterine bleeding (AUB), which encompasses both menstrual flow outside of patient-perceived normal volume, duration, regularity, or frequency and nonmenstrual vaginal bleeding, is a common gynecologic complaint, representing one-third of ambulatory gynecology visits.[1] Among peri- and postmenopausal women, AUB accounts for 70% of gynecology consultations.[1] Recognition and accurate diagnosis are essential for appropriate, timely management of AUB. Thus, evaluation and management of AUB are integral parts of the medical and surgical curriculum for OB/GYN clerkship students, as outlined by Association of Professors of Gynecology and Obstetrics (APGO) learning objectives.[2]

The differential diagnosis for AUB among reproductive-age women is broad and summarized by the widely accepted

Copyright © 2022 Adkins et al. This is an open-access publication distributed under the terms of the Creative Commons Attribution license.

PALM-COEIN (polyp, adenomyosis, leiomyoma, malignancy and hyperplasia, coagulopathy, ovulatory dysfunction, endometrial, iatrogenic, and not yet classified) nomenclature system developed by the International Federation of Gynecology and Obstetrics.[3] Care of women with AUB requires a systematic yet individualized approach for the provision of safe and comprehensive care. Thus, when presented with a case of AUB, clinicians must possess a wide array of existing knowledge to best approach and investigate the plethora of potential diagnoses and correctly treat and take care of the patient.

Given the sensitive nature of common gynecologic complaints and the potentially multistep workup and management plan, eliciting a focused history and developing differential diagnoses and a management plan pertaining to AUB can be challenging and anxiety provoking for learners. To promote provider comfort and precision with the evaluation of a common yet sensitive gynecologic complaint, there is a role for practice patient interviews with individualized feedback.

To facilitate an interactive educational approach to the evaluation of AUB, a formative standardized patient (SP) case was developed and implemented among second-year OB/GYN clerkship students. This encounter was implemented alongside another SP case on dyspareunia, aimed at bridging the gap within medical education regarding a common sexual complaint and students' sexual history-taking skills.[4] Given the stark differences between the cases and the potential of each to offer its own unique value, the SP encounters have been presented for publication separately.

The purpose of this SP case was to evaluate core clinical competencies (e.g., history and physical, differential diagnosis, initial evaluation, and workup), interpersonal communication, and professionalism via evaluation of an SP presenting with AUB. Second-year students participating in the OB/GYN clerkship at the Duke University School of Medicine were provided with the opportunity to participate in this SP encounter and utilize the skills obtained and feedback provided throughout the remainder of their rotation and beyond.

Methods

Target Audience

Second-year medical students at the Duke University School of Medicine participated in a 6-week clerkship in the OB/GYN department. Each student participated in three subrotations of 2 weeks duration that included an obstetric experience (e.g., labor and delivery), a gynecology experience (e.g., benign gynecology, female pelvic medicine and reconstructive surgery, gynecologic oncology), and an outpatient experience (e.g., prenatal care clinics, gynecology clinics, health department clinics). During the clerkship, students received weekly didactic lectures in case-based format from faculty members in the OB/GYN department. On the third week of the clerkship, students participated in two 30-minute SP encounters in the simulation center during scheduled didactic time. One encounter focused on dyspareunia and the other on AUB. The methodologies used to implement both SP encounters were similar; however, this publication describes the implementation tools used for the AUB SP encounter. Outside of three OB/GYN-based lectures presented during the students' first year of medical school and their having completed one full subrotation in the clerkship, students received no formalized preparation prior to the SP experience. They were highly encouraged to prepare for the encounters by consistent self-directed study throughout their OB/GYN clerkship and to utilize the APGO medical student objectives to ensure focus on the most clinically relevant material geared towards student education.[2]

The simulation experience was recorded and evaluated by residents and faculty members in the OB/GYN department in order to provide formative feedback for medical student participants. Students were instructed that they would participate in two SP encounters during the orientation on the first day of the clinical clerkship. Ten students per session day completed both of the SP encounters, which required 1 hour of scheduled time per student within the simulation center.

SP Preparation

We trained three SPs for the AUB case. SPs were provided ahead of time with information detailing their role during the encounter, including specific clinical history, physical exam findings, dress code, and positioning (Appendix A). Two SPs were present during each simulation session, allowing two students to move through the exercise at one time. Each case was scheduled for 30 minutes, with 15 minutes dedicated towards the direct patient encounter, 10 minutes for students to write the after-visit note, and a 5-minute break. The SP was positioned as seated on the exam room table, wearing a closed gown, with access to a sheet for draping. One physicial exam findings card was used in this case to describe the results of the pelvic exam. More information can be found in Appendix A. Specific details of the SP preparation for the dyspareunia case were provided by Hagey et al.[4]

Patient Encounter

Prior to the SP encounter, medical students were provided with basic patient information (e.g., name, age, clinical setting, chief complaint) and vital signs in the form of a door card (Appendix B). The door card included the allotted time for each activity and

Copyright © 2022 Adkins et al. This is an open-access publication distributed under the terms of the Creative Commons Attribution license.

specific tasks for the SP encounter (Appendix B). Each student was allotted 15 minutes to obtain a problem-focused history, perform a physical exam, discuss the next best workup and management steps, and address any questions from the patient. Within the exam room, students were notified when they had 5 minutes remaining and when the full 15 minutes were over. In the remaining 10 minutes, students completed a postencounter note including their medical decision-making, differential diagnosis, and proposed workup and management plan (Appendix C). During this 10-minute time period, the SP completed a standardized evaluation of the medical student (Appendix D). Videotaping from the SP encounter and corresponding documentation (student postencounter note and SP evaluation) were uploaded to Learning Space (a secure web-based simulation center management tool [CAE Healthcare]) immediately following the encounter.

After completion of the SP encounter, students were given access to the videotaped recording via a secure online portal. They were instructed to watch their encounter and to reflect on and evaluate their history-taking, physical exam, and patient counseling skills. Students were also provided with a video of a resident performing the case so that they could observe the history taking, differential diagnoses discussion, and counseling performed by an expert. Programs that do not have videotaping capacity can omit this portion of the exercise.

Learner Assessment

For each patient encounter, medical students received written feedback from SPs regarding their history-taking skills and physical exam. Numerical grades were calculated based on the postencounter documentation (e.g., written note, differential diagnosis, and management plan). The postencounter note was evaluated for inclusion of specific components of the history and physical, correct identification with supporting evidence of up to five potential diagnoses, and management options (Appendix C). Video recordings of the encounters were evaluated by residents and faculty in the OB/GYN department. Students reviewed their scores to assess how they had performed quantitatively.

Medical students received quantitative evaluation on their ability to perform a thorough evaluation of AUB and qualitative formative feedback on interpersonal skills during the encounter (Appendix D). Formative feedback by narrative comments from the SP was used as part of clerkship midpoint feedback, with which the clerkship director could help the student reflect on their strengths and weaknesses, and to set goals for the remainder of the clinical rotation. The categories evaluated included the student's introduction, history-taking skills, ability to elicit the patient's perspective, verbal and nonverbal communication, and ability to express empathy and respect towards the SP, as well as the effectiveness of the student's closure. Students were rated on a 5-point Likert scale (1 = *poor,* 2 = *fair,* 3 = *adequate,* 4 = *very good,* 5 = *excellent*; Appendix D). Numerical grades for the SP evaluation were determined using the aforementioned criteria. The purpose of all numerical grades included in the SP encounter was for students to get a sense of how they scored in relation to their colleagues and to readily identify areas for improvement. Numerical evaluations did not influence final grades for the OB/GYN clerkship.

Statistical Analysis

Deidentified data from all 2018-2019 Duke University OB/GYN second-year clerkship medical students who participated in both SP encounters were obtained from the clerkship coordinator and included for analysis. Demographic information included medical student gender and race, timing of clerkship within the academic year (tertiles), subrotation clinical score, mean standardized examination performance (raw score and national percentile), and overall clerkship score (fail, pass, high pass, or honors). Additionally, numeric data from postencounter student notes, postencounter SP evaluations, and video evaluation specific to the AUB patient encounter were included in the analysis. Summary statistics of continuous (median) and categorical variables (percentage) were calculated.

Qualitative feedback regarding both SP encounters from student participants was also included in the analysis. The undergraduate medical education team of the Duke University School of Medicine routinely compiled midpoint and end-of-clerkship feedback (Appendix E). Students were given the opportunity to provide open-ended feedback on their experience as a learner on this clerkship and to rate how well the SP encounters enhanced their overall learning. Results regarding the SP encounters were compared with other academic experiences held during the clinical rotation. These results were compiled in aggregate and released every 6 months to clerkship leadership to allow for deidentification of individual feedback.

Results

Student Characteristics

Both SP cases were implemented with 101 second-year medical students at the Duke University School of Medicine. A majority of the participants self-identified as female (52%) and White (63%). More information regarding student characteristics and clerkship performance is available in Table 1.

Copyright © 2022 Adkins et al. This is an open-access publication distributed under the terms of the Creative Commons Attribution license.

Table 1. Characteristics of Student Participants in the Standardized Patient Encounter (*N* = 101)

Characteristic	No. (%)	*M* (*SD*)
Gender		
Female	52 (51)	
Male	49 (49)	
Race		
African American or Black	4 (4)	
Asian	30 (30)	
White	64 (63)	
Other	1 (1)	
Nondisclosed	2 (2)	
Timing of OB/GYN clerkship		
Rotation 8/18-12/18	37 (37)	
Rotation 1/19-4/19	34 (34)	
Rotation 4/19-8/19	30 (30)	
NBME raw score		81.5 (6.2)
NBME national percentile		65% (25%)
Clerkship grade		87% (9%)
Clerkship overall score		
Honors	58 (57)	
High pass	34 (34)	
Pass	7 (7)	
Fail	0 (0)	
Incomplete	2 (2)	

Patient Interaction and History-Taking Skills

As it pertains to the AUB case, on average, students were rated by SPs as adequate history takers across all categories evaluated, with a mean overall score of 3.45 (*SD* = 0.15) out of 5. Compared with any other category, more students (*n* = 69, 68%) were rated as very good or excellent with regard to respect (i.e., students demonstrated an attitude of acceptance, established partnership, and performed the physical exam with great sensitivity to pain and modesty). However, more students appeared to have struggled with closing the encounter, as only 38% (*n* = 38) were rated as very good or excellent in this category (Table 2).

Out of the 11 components of the history regarding the AUB case, the most common aspects that were elicited regarded symptom onset and duration (*n* = 108, 99%) and symptom severity (i.e., quality of life; *n* = 96, 88%). Fewer than one in five students inquired about the patient's last menstrual period (*n* = 19, 17%), and only 44 (40%) reviewed any current medications.

Table 2. Mean Scores for Each Category of Learners' Interpersonal Skills Evaluated by the Standardized Patient (*N* = 101)

Evaluation Category[a]	*M* (*SD*)
Introduction	3.4 (0.7)
Questioning skills	3.5 (0.6)
Elicit patient perspective	3.4 (0.7)
Verbal communication	3.5 (0.7)
Nonverbal communication	3.4 (0.6)
Empathy	3.4 (0.8)
Respect	3.7 (0.6)
Closure	3.1 (1.0)

[a]Rated on a 5-point scale (1 = *poor*, 2 = *fair*, 3 = *adequate*, 4 = *very good*, 5 = *excellent*).

Differential Diagnosis and Workup

Of the five diagnoses to consider from the AUB SP encounter, the vast majority of students (*n* = 95, 94%) correctly identified at least one possible diagnosis and correctly provided evidence following the identification. Less than half (*n* = 45, 44%) correctly identified two diagnoses supported with evidence, and only one in four identified three diagnoses supported with evidence. Of note, any student who provided a potential diagnosis (e.g., uterine fibroid) but did not provide evidence for why they suspected the diagnosis (e.g., irregular uterine shape on exam) did not receive credit for correctly providing evidence following diagnosis identification. The most likely potential diagnoses identified by students were endometrial cancer and/or hyperplasia (*n* = 64, 63%) and uterine leiomyoma (*n* = 61, 60%; Table 3). Almost all students (*n* = 99, 98%) requested a pelvic exam as a part of their evaluation, and a majority (*n* = 82, 81%) desired to obtain a pelvic ultrasound as a part of their initial management and workup plan (Table 4).

Student Feedback

Eighty-two students (81%) completed the end-of-clerkship evaluation, which provided cumulative feedback for both SP encounters. Overall, students positively discussed both of the SP encounters and enjoyed being able to receive formative feedback on their clinical performance in an environment that did not impact their overall clerkship grade.

A total of 47 students (57%) indicated that the SP experience enhanced their overall learning on this clerkship at least adequately well or better. As it related to educational value, the SP experience proved similarly valuable, with 75% of students rating the educational value as average, above average, or excellent. Lastly, in a free-response section of the end-of-clerkship evaluation, two students mentioned the SP encounters as the most valuable aspect of the clerkship.

Table 3. Abnormal Uterine Bleeding Differential Diagnosis on Postencounter Learner Note (*N* = 101)

	No. (%)	
Proposed Diagnosis	Correctly Identified	Correctly Provided Evidence After Identifying Diagnosis[a]
Endometrial cancer/hyperplasia	64 (64)	63 (98)
Leiomyoma	61 (60)	61 (100)
Adenomyosis	30 (30)	30 (100)
Ovulatory dysfunction	22 (22)	20 (91)
Endometrial polyp	18 (18)	18 (100)
None correct	6 (6)	

[a]Number and percentage in this column are based on total number correctly identified.

Copyright © 2022 Adkins et al. This is an open-access publication distributed under the terms of the Creative Commons Attribution license.

Table 4. Workup of Abnormal Uterine Bleeding on Postencounter Learner Note ($N = 101$)

Requested Test/Exam	No. (%)
Pelvic exam	99 (98)
Pelvic ultrasound	82 (81)
Complete blood count	52 (51)
Endometrial biopsy	35 (35)
Bhcg	26 (26)
Coagulation panel	8 (8)
Thyroid panel	6 (6)
Cervical cultures	5 (5)

Discussion

We have described the successful implementation of an SP case on AUB at a single medical institution during the OB/GYN clerkship for medical students. This encounter was implemented alongside another encounter focused on dyspareunia, which used similar implementation methodology and methods of student evaluation, with the exception of SP historical details specific to AUB (Appendix A).[4] Overall, our students adequately demonstrated interpersonal history-taking skills that allowed them to elicit pertinent medical information relating to AUB. For the majority of students, the information garnered led to the correct identification of at least one potential diagnosis with supporting evidence and a correct next step for management or evaluation.

The potential diagnoses that encompass the commonly utilized PALM-COEIN nomenclature system when evaluating patients with AUB include a wide range of diagnoses from benign causes to life-threatening illnesses. The most common diagnosis correctly identified by our students was endometrial cancer or hyperplasia (63%), an important diagnosis within the field of OB/GYN representing the most common gynecologic malignancy.[2] Survey studies investigating US medical students' perspectives on oncology education have reported OB/GYN as being among the top clinical clerkships to incorporate the most didactic time towards oncology teaching, as well as incorporating the highest percentage of patients with cancer in medical students' care.[5,6] The commonality of uterine hyperplasia or carcinoma among OB/GYN patients and the potential for emphasis on oncology teaching may be reasons why a majority of our students identified uterine carcinoma as a potential diagnosis for this case. At our medical institution, the ability to identify critical diagnoses (e.g., carcinoma) is a highly valued skill to cultivate throughout medical education. This diagnostic skill is later evaluated through a final objective structured clinical examination (OSCE)—a performance-based evaluation of clinical skills usually administered at the end of the clinical year—that is hosted by 151 of 154 medical schools within the USA.[7] Thus, implementing an SP encounter, such as the AUB case, that allows students to exercise their diagnostic abilities of critically important pathologies outside of the OSCE examination may be beneficial for students as they prepare for the exam later within the clinical year.

Additionally, a majority of our students displayed very good or excellent interpersonal skills, such as respect, throughout the patient encounter; however, students struggled most in their ability to properly close the encounter. This observation is not unique to our cohort. Students' lack of confidence and minimal formal training in patient counseling have been described as factors that can influence their ability to counsel patients and close an encounter in a more effective manner.[8-10] Additionally, SP feedback suggests that this situation occurs because students end the encounter with a promise to return after talking to the team about the patient as opposed to making a plan or counseling the patient themselves. Further research is needed to better understand the barriers students face while closing patient encounters, counseling patients, and providing next steps for evaluation and management.

It is important to note the limitations of our study. This SP case has been implemented and evaluated at only a single medical center, which may limit generalizability. This SP encounter was implemented alongside another SP encounter evaluating dyspareunia.[4] Thus, the feedback regarding the SP experience from the student perspective was directed towards both patient encounters and not evaluated separately. In addition, our medical center contains a well-organized clinical skills lab and resources such as LearningSpace, a secure web-based simulation center management tool for compiling, scoring, and reviewing medical student and SP notes, as well as videos related to the SP encounter. For institutions without similar technological resources, in-person or written feedback could be given directly to students following the encounter. Completion of patient notes could be performed by use of a word-processing system and sent electronically to resident and faculty evaluators, while feedback could be returned to students in person or written out. An additional limitation regarding the performance of students within our cohort is the 30-minute time constraint, which could have impacted their ability to fully elicit a patient history and/or document their full encounter. However, given the similarities to a real patient encounter and the potential to prepare medical students for the end-of-the-year OSCE exam at the Duke School of Medicine, we opted to keep this exercise consistent with similar OSCE requirements.

In conclusion, this SP case effectively provided students with the opportunity to exercise their diagnostic and management

Copyright © 2022 Adkins et al. This is an open-access publication distributed under the terms of the Creative Commons Attribution license.

skills when faced with a patient with a common gynecologic complaint. Given the broad list of potential diagnoses for AUB, it is imperative that learners encompass a wide range of existing knowledge in order to arrive at the correct diagnosis and management plan after eliciting pertinent information from the medical history. It was reassuring to see that a majority of our students correctly identified at least one potential diagnosis and ranked the SP encounters similarly in value amongst other academic clerkship activities.

Appendices

A. SP Information.docx

B. Learner Information.docx

C. Postencounter Learner Note.docx

D. Postencounter SP Evaluation.docx

E. Learner End-of-Clerkship Feedback.docx

All appendices are peer reviewed as integral parts of the Original Publication.

LaMani D. Adkins: Fourth-Year Medical Student, Duke University School of Medicine; ORCID: https://orcid.org/0000-0003-4698-9283

Benjamin S. Harris, MD, MPH: Clinical Fellow, Division of Reproductive Endocrinology and Infertility, Department of Obstetrics and Gynecology, Duke University Medical Center

Cescille Gesher: Program Coordinator, Department of Obstetrics and Gynecology, Duke University Medical Center

Tracey Reynolds: Assistant SP Trainer and External Client Coordinator of the Clinical Skill Program, Office of Curricular Affairs, Duke University School of Medicine

Kelly Branford: Director of the Clinical Skills Program, Office of Curricular Affairs, Duke University School of Medicine

Melody Baldwin, MD, MPH: Assistant Professor of Obstetrics and Gynecology, Department of Obstetrics and Gynecology, Duke University Medical Center

Sarah Dotters-Katz, MD, MMHPE: Director of Undergraduate Medical Education and Associate Professor of Obstetrics and Gynecology, Department of Obstetrics and Gynecology, Duke University Medical Center

Disclosures
None to report.

Funding/Support
None to report.

Ethical Approval
Reported as not applicable.

References

1. Spencer CP, Whitehead MI. Endometrial assessment re-visited. *Br J Obstet Gynaecol*. 1999;106(7):623-632. https://doi.org/10.1111/j.1471-0528.1999.tb08358.x
2. *APGO Medical Student Educational Objectives*. 11th ed. Association of Professors of Gynecology and Obstetrics; 2019.
3. Munro MG, Critchley HOD, Broder MS, Fraser IS; FIGO Working Group on Menstrual Disorders. FIGO classification system (PALM-COEIN) for causes of abnormal uterine bleeding in nongravid women of reproductive age. *Int J Gynaecol Obstet*. 2011;113(1):3-13. https://doi.org/10.1016/j.ijgo.2010.11.011
4. Hagey JM, Toole J, Branford K, Reynolds T, Livingston E, Dotters-Katz SK. Understanding sexual complaints and history taking: a standardized patient case on dyspareunia for obstetrics and gynecology clerkship students. *MedEdPORTAL* 2020;16:11001. https://doi.org/10.15766/mep_2374-8265.11001
5. Mattes MD, Patel KR, Burt LM, Hirsch AE. A nationwide medical student assessment of oncology education. *J Cancer Educ*. 2016;31(4):679-686. https://doi.org/10.1007/s13187-015-0872-6
6. Neeley BC, Golden DW, Brower JV, Braunstein SE, Hirsch AE, Mattes MD. Student perspectives on oncology curricula at United States medical schools. *J Cancer Educ*. 2019;34(1):56-58. https://doi.org/10.1007/s13187-017-1265-9
7. Curriculum Reports: assessment methods in clinical clerkship experiences (formative and/or summative). *Association of American Medical Colleges*. Accessed April 10, 2021. https://www.aamc.org/data-reports/curriculum-reports/interactive-data/assessment-methods-clinical-clerkship-experiences-formative-and/or-summative
8. McCord EC, Smorowski-Garcia K, Doughty A. Assessment at one school of students' abilities and confidence in diabetic patients' education. *Acad Med.* 1997;72(12):1116-1118. https://doi.org/10.1097/00001888-199712000-00027
9. Williams AS, Patel PM, Beucke NL, Koopman RJ. Community-based medical student nutrition counseling training for low-income families. *PRiMER*. 2018;2:5. https://doi.org/10.22454/PRiMER.2018.809708
10. Park KY, Park HK, Hwang HS. Group randomized trial of teaching tobacco-cessation counseling to senior medical students: a peer role-play module versus a standardized patient module. *BMC Med Educ*. 2019;19:231. https://doi.org/10.1186/s12909-019-1668-x

Received: June 2, 2021
Accepted: October 27, 2021
Published: January 28, 2022

Copyright © 2022 Adkins et al. This is an open-access publication distributed under the terms of the Creative Commons Attribution license.
